# How Widespread Are the “Young” Neurons of the Mammalian Brain?

**DOI:** 10.3389/fnins.2022.918616

**Published:** 2022-06-06

**Authors:** Marco Ghibaudi, Luca Bonfanti

**Affiliations:** ^1^Neuroscience Institute Cavalieri Ottolenghi (NICO), Orbassano, Italy; ^2^Department of Veterinary Sciences, University of Turin, Grugliasco, Italy

**Keywords:** adult neurogenesis, brain plasticity, comparative neuroplasticity, immature neurons, doublecortin, subcortical regions

## Abstract

After the discovery of adult neurogenesis (stem cell-driven production of new neuronal elements), it is conceivable to find young, undifferentiated neurons mixed with mature neurons in the neural networks of the adult mammalian brain. This “canonical” neurogenesis is restricted to small stem cell niches persisting from embryonic germinal layers, yet, the genesis of new neurons has also been reported in various parenchymal brain regions. Whichever the process involved, several populations of “young” neurons can be found at different locations of the brain. Across the years, further complexity emerged: (i) molecules of immaturity can also be expressed by non-dividing cells born during embryogenesis, then maintaining immature features later on; (ii) remarkable interspecies differences exist concerning the types, location, amount of undifferentiated neurons; (iii) re-expression of immaturity can occur in aging (dematuration). These twists are introducing a somewhat different definition of neurogenesis than normally assumed, in which our knowledge of the “young” neurons is less sharp. In this emerging complexity, there is a need for complete mapping of the different “types” of young neurons, considering their role in postnatal development, plasticity, functioning, and interspecies differences. Several important aspects are at stake: the possible role(s) that the young neurons may play in maintaining brain efficiency and in prevention/repair of neurological disorders; nonetheless, the correct translation of results obtained from laboratory rodents. Hence, the open question is: how many types of undifferentiated neurons do exist in the brain, and how widespread are they?

## Introduction

To perform its functions the brain needs stability, in terms of organized (genetically determined) neural networks assuring proper neuronal communication. Neural elements assemble into relatively stable neural circuits after embryonic neurogenesis and early life. Their maturation occurs with remarkable regional differences, leaving spots of immaturity that mostly fade with age, yet, at some locations, continue throughout life. All the exceptions by which definitive stability is not reached (at the synapse, nerve cell/processes, neural network level) do represent plasticity, allowing postnatal structural changes in the system, driven by experience. Even neurogenesis can take place in the adult mammalian brain. It can be viewed as an odd exception to the complex, genetically determined structure of the brain, with its billions of neurons and trillions of synaptic contacts (Chklovskii et al., 2004). The new neurons born in stem cell niches and integrating into pre-existing neural circuits, generated controversies for decades due to a mix of conterintuitive novelty and objective technical difficulties (Kaplan, 2001). Then, a sort of confusion continued (and continues) to characterize this fascinating research field (Lipp and Bonfanti, 2016; Parolisi et al., 2018; Duque and Spector, 2019; Petrik and Encinas, 2019; Bonfanti and Seki, 2021; Duque et al., 2022), especially concerning its existence/rate in adult humans (Sanai et al., 2011; Moreno-Jiménez et al., 2021; Sorrells et al., 2021) and the putative occurrence of non-canonical neurogenesis within parenchymal brain regions (Bonfanti and Peretto, 2011; Feliciano et al., 2015). A major unresolved point is the occurrence of “young,” immature neurons in the adult brain, in the absence or very low levels of cell division (Moreno-Jimenéz et al., 2019; Sorrells et al., 2018, 2019). After decades of investigations, many aspects of the neurogenic processes have not been fully grasped, and further elements of novelty might exist (Benedetti et al., 2020; La Rosa et al., 2020a; Urbán, 2022). An extreme heterogeneity of types, forms and nuances of brain structural plasticity is emerging, ultimately providing the brain with populations of “young” neurons (Bonfanti and Charvet, 2021). In this growing complexity, the question should be asked whether the mix of results reported in the literature depends on real differences in types of plasticity, interspecies differences, tissue fixation procedures, technical pitfalls, and/or different interpretations from the Authors.

## Identifying the “Young” Neurons: A Tricky Task

Neurodevelopmental studies are accompanied by the use of cell markers: molecules that are (or should be) expressed by specific populations of cells, indicating their belonging to defined categories in the dynamic process of neuronal specification and maturation (stem cells, neural progenitors, immature, maturing, and mature neurons; Kempermann et al., 2004; Von Bohlen Und Halbach, 2007; Urbán and Guillemot, 2014; Sarnat, 2015; Bonfanti and Charvet, 2021). Yet, here come some difficulties, linked to a series of facts: (i) neuronal maturation is a multistep process consisting of gradients of molecular expression through time, sometimes making it nebulous to sharply define stages (Kempermann et al., 2004; Sarnat, 2015); (ii) the time period corresponding to the phase of immaturity can remarkably vary across cell populations and brain regions (La Rosa et al., 2020a); (iii) as a consequence, different types of “young” neurons can exist and co-exist in different animal ages/brain regions, as an expression of different forms of plasticity (Bonfanti and Charvet, 2021); (iv) it is becoming more and more evident that these aspects can remarkably vary depending on the animal species considered, with increasing divergence when comparing small-sized, lissencephalic, and large-sized, gyrencephalic brains (Kohler et al., 2011; Brus et al., 2013; Paredes et al., 2016a; La Rosa et al., 2020b; Franjic et al., 2022; Schmitz et al., 2022).

To give examples, the markers for neuronal immaturity doublecortin (DCX) and polysialylated neural cell adhesion molecule (PSA-NCAM) were unanimously considered good indicators for neurogenesis and cell migration (Brown et al., 2003; Bonfanti, 2006). Detection of DCX was often used to “map” neurogenic processes, and its expression was considered a proxy for newborn neurons. Nevertheless, while this statement can be true in neurogenic niches (Brown et al., 2003; but see below), it is not for the so-called “non-newly generated, immature” neurons of the cerebral cortex (Gómez-Climent et al., 2008; Piumatti et al., 2018; Rotheneichner et al., 2018; La Rosa et al., 2020a; see below). Recent techniques allowing to go far beyond the simple localization of cell markers, such as clonal lineage tracing and single-cell transcriptomic profiling, confirm the existence of remarkable heterogeneity in cell populations and across species (Shohayeb et al., 2021; Franjic et al., 2022; Schmitz et al., 2022). Finally, a further kind of re-expression of immaturity molecules, in which neurons dedifferentiate to a pseudo-immature status (“dematuration”), has been shown to occur in aging, inflammation, and hyperexcitation (Hagihara et al., 2019). On the whole, the benefit of using markers appears more fuzzy than previously thought.

Despite technical and theoretical difficulties, researchers agree that the mammalian brain does contain populations of “young” neurons, which might be extremely interesting in the perspective of a full understanding of brain development/maturation, as well as for preventive/therapeutic approaches for neurological disorders. It is more and more evident that we cannot mix all these cells in the same cauldron; we need to know more about their features, origin, location, fate, role in plasticity.

## Are There Different Types of “Young” Neurons?

Before the revolution of adult neurogenesis, the classic view of the mammalian brain was that of a “non-renewable” tissue, in which the only structural changes allowed consisted of formation/elimination of synaptic contacts (synaptic plasticity). In that view, after the end of embryonic neurogenesis, the brain was composed of mature neurons having only the possibility to change microscopically at the tip of their dendritic/axonal processes, or in some cases, to regenerate axonal/dendritic portions (Magee and Grienberger, 2020). Adult neurogenesis makes it possible the production of new neurons starting from neural stem cell “niches” (Doetsch et al., 1999), involving processes of cell division, specification, differentiation (in some cases migration), maturation, and final integration into the pre-existing neural circuits (Kempermann et al., 2004; Aimone et al., 2014; Bond et al., 2015; Lim and Alvarez-Buylla, 2016). Thousands of studies carried out mostly in rodents have unraveled the molecular and cellular mechanisms of stem cell-driven neurogenesis in the two main neurogenic sites: the ventricular-subventricular zone of the forebrain lateral ventricles (V-SVZ; Lim and Alvarez-Buylla, 2016) and the subgranular zone of the dentate gyrus in the hippocampus (SGZ; Kempermann et al., 2015). Nevertheless, even these “canonical” neurogenic processes are characterized by heterogeneity: (i) they are abundant and widespread in the whole nervous system of non-mammalian vertebrates, while highly restricted in mammals (Bonfanti, 2011; Lindsey et al., 2018; Lange and Brand, 2020); (ii) their extension in the animal lifespan strongly depends on the species (Charvet and Finlay, 2018; Snyder, 2019; Bonfanti and Charvet, 2021), with dramatic region-specific reduction in different regions and animal groups (e.g., the SVZ in humans, Sanai et al., 2011; both neurogenic sites in dolphins; Patzke et al., 2015; Parolisi et al., 2017, 2018); (iii) the maturational times of the newlyborn neurons can remarkably vary, spanning from 3 to 4 weeks in rodents to 6 months in monkeys (Kohler et al., 2011).

**FIGURE 1:**
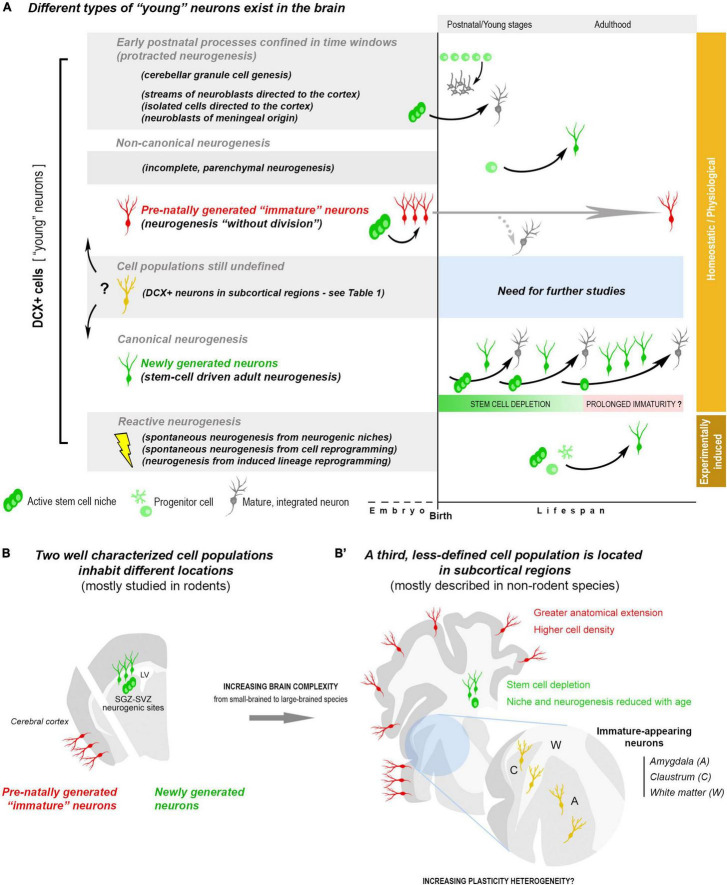
Summary of the heterogeneity of “young,” undifferentiated neurons existing in the postnatal and adult mammalian brain, on the basis of the current knowledge. **(A)** Young neurons (e.g., DCX^+^ cells) can belong to different cell populations, with different origin/fate, likely displaying different types of plasticity: postnatal streams of neuroblasts and postnatal genesis of neuronal populations; adult canonical and non-canonical neurogenic processes. Most of the young neurons can be considered as physiological/homeostatic events, others are reactive neurogenesis to lesion/disease (orange and brown on the right). Two cell populations/processes (in the white background) have driven much interest because of their origin/outcome and, also, being at the basis of some confusions: newly generated neurons, produced by division of stem/progenitor cells (canonical adult neurogenesis, green cells), and non-newly generated (dormant) “immature” neurons, which are generated pre-natally, then persisting in an immature state (neurogenesis “without division,” red cells). In canonical neurogenesis (bottom), is represented a hypothesis for the persistence of immature, slowly maturing neurons after depletion of the stem cell reservoir. **(B)** The two populations of young neurons mentioned above (newly generated and non-newly generated “immature” neurons) inhabit different brain regions: the forebrain/hippocampal neurogenic sites and the cerebral cortex layer II. Their anatomical distribution and amount appear to differ among mammals **(B,B’)**, with a prevalence of stem cell-driven neurogenesis in small-brained species and a greater abundance of “dormant” neurons in large-brained ones, extending to neocortex (La Rosa et al., 2020b). A third, less studied population (yellow cells), also expressing DCX, has been described in subcortical regions of different mammals (see Table 1). Here they are indicated as immature-appearing neurons since it is not clear whether they are composed of either “dormant” neurons, newlyborn neurons, or both.

Another source of young neurons is represented by “protracted” neurogenesis: streams of neuroblasts or isolated cells generated during late embryogenesis and migrating into the postnatal cortex (Le Magueresse et al., 2011, 2012; Riccio et al., 2012; Paredes et al., 2016b; Bifari et al., 2017; Nascimento et al., 2022). These processes show different origin and features in rodents and primates, likely due to different neurodevelopmental schedules in mammals (Workman et al., 2013; Bonfanti and Charvet, 2021). They seem particularly prominent in human infants, delivering inhibitory interneurons into the frontal and temporal lobes (Paredes et al., 2016b; Nascimento et al., 2022). This delayed addition of neurons can enrich the young neural circuits with new elements while they are highly plastic and modifiable by early life experiences. Similarly, the entire population of cerebellar granule cells is added postnatally, yet, in this case involving multiple rounds of proliferation of progenitor cells forming a transient, subpial germinative layer, then undergoing exhaustion at specific postnatal ages (Altman and Bayer, 1997; Figure 1A).

**TABLE 1:** Detection of young, undifferentiated neurons in subcortical brain regions of mammals: interspecies differences and heterogeneous interpretation in the literature.

Brain region	Animal species	Age	Proposed nature		References
			Physiological condition	Experimental condition	
AMYGDALA	Mouse (*Mus musculus*)	All ages	No immature cells detectable		(Mostly unpublished negative results and personal observations)
		7–12 weeks		Newly generated (YFP transgenic mice, neurosphere assay) (BrdU and DCX)	Jhaveri et al., 2018
	Rabbit (*Orictolagus cuniculus*)	3–6 months	Chains of immature neurons (DCX, PSA-NCAM), with a few newly generated elements (BrdU 40 mg/kg)		Luzzati et al., 2003
	Sheep (*Ovis aries*)	1 week 4 months 2 years	Pre-natally generated (BrdU 20 mg/kg, injected during pregnancy and in adulthood; confocal microscopy)		Piumatti et al., 2018
	Marmoset (*Callithrix jacchus*)	4 years	Newly generated (PSA-NCAM expression) (BrdU 200 mg/kg, light microscopy)		Marlatt et al., 2011
	Squirrel monkey (*Saimiri sciureus*) Cynomolgus monkeys (*Macaca fascicularis*)	3–6 years 6–12 years	Newly generated (PSA-NCAM expression and other markers) (BrdU 50 mg/kg twice a day for 3 days)		Bernier et al., 2002
	Macaque (*Macaca mulatta*)	1 day–9,5 years		Immature neurons - Cell migration suggested (Bcl-2 expression) [*hippocampal lesion*]	Chareyron et al., 2021
		12 years 21 years 31 years	Immature neurons (DCX and PSA-NCAM expression)		Zhang et al., 2009
	Macaque (*Macaca fascicularis, Macaca nemestrina*)	2,5 years	Immature neurons (DCX and PSA-NCAM expression)		Fudge et al., 2012
	Human (*Homo sapiens*)		Immature neurons (expression of PSA-NCAM but not Ki67)		Martí-Mengual et al., 2013
	Human (*Homo sapiens*)	From embryo to adult	Immature neurons until adolescence, then possibly undergoing maturation		Sorrells et al., 2019
	Mouse, Rat, Human	7 weeks (M) 2–4 months (R) 18–65 years (H)	Relation with behavior (DCX expression: protein isolation; RNAseq, qRT-PCR)		Maheu et al., 2021
CLAUSTRUM	Sheep (*Ovis aries*)	1 week 4 months 2 years	Pre-natally generated (BrdU 20 mg/kg, injected during pregnancy and in adulthood; confocal microscopy)		Piumatti et al., 2018
WHITE MATTER	Rabbit (*Orictolagus cuniculus*)	3–6 months	Chains of PSA-NCAM + cells		Luzzati et al., 2003
	Macaque and Human (*Macaca mulatta Homo sapiens*)	2 weeks–12 years (M) 6 weeks–49 years (H)	Migrating cells (DCX expression, DCX mRNA) Decreased during infancy	Decreased in schizofrenic patients *[schizofrenia]*	Fung et al., 2011
	Sheep (*Ovis aries*)	1 week 4 months 2 years	Clusters of immature neurons (non-newly generated in sheep; not coexpressing Ki67 in dolphin)		La Rosa et al., 2018
	Dolphin (*Tursiops truncatus Stenella coeruloalba*)	1–9 days to adult			

By using markers typically expressed in newlyborn neurons, it was suggested that other cell populations located in various “parenchymal” regions (outside the neurogenic stem cell niches) could also be potentially newly generated. In some cases, by using markers of cell division (Ki-67 antigen) and pulse-labeling trace-tracing with tymidine analog (BrdU), the newlyborn nature of the “young” cells was proven (Dayer et al., 2005; Kokoeva et al., 2005; Luzzati et al., 2006; Ponti et al., 2006, 2008). Yet, substantial differences exist between parenchymal neurogenesis and canonical neurogenic sites: (i) most of these processes do not start from a morphologically-defined, constitutively-active stem cell niche and/or are not followed by long-term cell survival and functional integration into the pre-existing circuits (so-called “incomplete neurogenesis”; Bonfanti and Peretto, 2011); (ii) some processes are detectable only in some mammalian species, being not present in rodents (Luzzati et al., 2006; Ponti et al., 2006, 2008); (iii) newborn neurons can be found in the brain parenchyma in different experimental conditions, both as lesion-induced, spontaneous “reactive” neurogenesis from the neurogenic sites (Arvidsson et al., 2002) or parenchymal astrocytes (Magnusson et al., 2014; Nato et al., 2015), and after induced lineage reprogramming of glial cells (Mattugini et al., 2019; Zamboni et al., 2020), including lineage conversion of oligodendrocytes precursors cells (Heinrich et al., 2014) and glia-to-neuron conversion in the hippocampus (Lentini et al., 2021).

In summary, it is more and more evident that different types of young, undifferentiated neurons, stepping away from “canonical” adult neurogenesis, do populate the postnatal and adult brain, possibly contributing to different aspects of its maturation, plasticity, and reaction to lesion/pathology. In this complexity, the simplistic belief that DCX^+^ cell detection is a proxy for neurogenesis should be replaced by more nuanced landscapes.

## Canonical and Non-Canonical Neurogenesis: From Neural Stem Cells to “Dormant” Immature Neurons

A novel, counterintuitive example of “young” neurons has been introduced by the demonstration that some populations of undifferentiated cells, displaying the same markers of immaturity expressed by newlyborn neurons, do not divide at all (Gómez-Climent et al., 2008; Piumatti et al., 2018). These cells were firstly described in the piriform cortex layer II (Seki and Arai, 1991; Bonfanti et al., 1992), and are currently (provisionally) referred to as “immature” or “dormant” neurons, since they are generated pre-natally, then remaining in a “standby” state of immaturity for long time (Luzzati et al., 2009; Gómez-Climent et al., 2008; Klempin et al., 2011; Bonfanti and Nacher, 2012; König et al., 2016; Rotheneichner et al., 2018; Benedetti and Couillard-Després, 2022). This idea of “young,” non-dividing neurons has evolved slowly through the years, somehow overwhelmed by the emphasis focused on adult neurogenesis (Bonfanti and Seki, 2021). These neurons can re-activate their maturational process to finally mature and integrate into adult circuits (Rotheneichner et al., 2018; Benedetti et al., 2020). This “neurogenesis without division” can be important for two reasons: first, because they can represent a reservoir of new elements in brain regions that are not endowed with stem/progenitor cells (e.g., the cerebral cortex; Rotheneichner et al., 2018; La Rosa et al., 2019, 2020b; Coviello et al., 2022); second, because it may explain why DCX^+^ cells can be found in non-neurogenic regions, in the absence of cell division. The molecular mechanisms responsible for the stop/re-start of the maturational process, as well as the role they can play once inserted in the circuits, are currently unexplored (Benedetti and Couillard-Després, 2022). Accordingly, “standby” mode neurons were previously described in the spinal cord (Marichal et al., 2009; Kempermann, 2012), thus showing that the puzzling nature of these immature cells can be a general principle in the mammalian nervous system.

For their features, the dormant neurons might be considered as part of the non-canonical neurogenesis, intended as different ways through which the brain can produce/activate new neurons in absence of stem cell division (Bonfanti and Nacher, 2012; Benedetti and Couillard-Després, 2022). The distinction between canonical and non-canonical neurogenesis is at present semantic (Feliciano et al., 2015), since it has been changed/adapted to new discoveries over the years and can evolve in the future, according to the level of heterogeneity of the processes. Non-canonical neurogenesis should include both parenchymal neurogenesis (involving cell division but appearing incomplete) and dormant neurons (not involving cell division after birth but appearing complete). Nevertheless, the final outcome can be the same in canonical adult neurogenesis and dormant neurons, since both processes lead to the addition of new neuronal elements in the neural circuits.

Considering the current knowledge, at least two neuronal populations can be placed into categories on the basis of their origin: the newly generated neurons of the SVZ and SGZ (canonical neurogenesis; extensively studied) and the non-newly generated, cortical “immature” neurons of the cerebral cortex layer II (prenatal origin; far less studied). An ill-defined landscape remains in the inner part of the hemispheres, with reference to populations of subcortical DCX^+^ neurons of uncertain origin (Figures 1B,B’).

## The Confusion Still Existing in Subcortical Regions

Brain subcortical regions, including gray (amygdala, claustrum) and white matter (external capsule, corpus callosum), are particularly enriched in “young” neurons, with interspecies differences. While a few (or even no) DCX^+^ neurons can be detected in the amygdala of mice, high amount of these cells has been described in non-human primates (Bernier et al., 2002; Zhang et al., 2009; Marlatt et al., 2011; Fudge et al., 2012; Chareyron et al., 2021) and humans (Martí-Mengual et al., 2013; Sorrells et al., 2019; Table 1). Interestingly, this situation seems to mirror what reported for the cerebral cortex (La Rosa et al., 2020b), thus suggesting that a widespread occurrence of “young” neurons in regions devoid of stem cell niches might be a general trend for non-rodent mammals with reduced canonical neurogenesis (Figures 1B,B’). At present, this is only an hypothesis that should be verified with systematic, quantitative interspecies analyses, similarly to those carried out in the cortex (La Rosa et al., 2020b). Very few is known regarding the real nature/origin of the subcortical DCX^+^ cells, sometimes interpreted as newly generated in the past (Table 1), when DCX was considered a proxy for neurogenesis (Bonfanti and Seki, 2021). Taking into account that such neurons seem to be important in primates (references in Table 1), the use of experimental approaches aimed at establishing their origin are far from easy. Nonetheless, it is worth mentioning that large populations of dividing glial cells, mostly oligodendrocyte precursors, are widely distributed in the whole brain parenchyma (Clayton and Tesar, 2021; Semënov, 2021), what can represent an important background noise for neurogenesis researchers. Due to the importance of white matter, amygdala and claustrum for proper brain connectivity and functioning, from emotions to conscience, a deeper knowledge of their “young” neurons is needed.

## Conclusion and Future Perspectives

The aim of this Perspective is to underline the extreme heterogeneity of neuroplasticity potentially deriving from different categories of young neurons that populate the mammalian brain with remarkable interspecies variation. The emerging landscape goes beyond the old vision of a “static” brain and also that, more recent, of canonical adult neurogenesis studied in rodents. Prominent postnatal streams of neuroblasts, high amount of “dormant” cortical neurons and unidentified DCX^+^, immature cells in subcortical regions appear to be a prevalent feature of young-adult gyrencephalic species (Paredes et al., 2016a,b; La Rosa et al., 2020b; Nascimento et al., 2022). The expanded primate brains show multifaceted elements of complexity, involving neuroanatomy, numbers of neurons, and amazing cell type distinctions (Franjic et al., 2022; Schmitz et al., 2022). The different populations of young neurons seem to follow such a complexity: dormant neurons extending in wide regions not endowed with stem cells; postnatal streams delivering inhibitory interneurons; immature neurons becoming principal, pyramidal elements; newlyborn hippocampal granules being excitatory neurons.

As frequently pointed out, relying exclusively on laboratory rodents comes with several costs that are often not considered, reducing the possibility for proper translation to humans (Brenowitz and Zakon, 2015; Faykoo-Martinez et al., 2017). We need to grasp whether, how, and to which extent, evolution has sculpted interspecies differences from mice to humans, in order to check the current hypothesis: mammals characterized by large brain size, high computational complexity, and long postnatal developmental periods, show lower levels of neurogenesis coexisting with higher content of “young” neurons, and this may represent an evolutionary choice.

By following the ideas presented here, immature/slowly maturing neurons might also persist in neurogenic sites of long-living species, granting the pursuing of plasticity even after stem cell depletion. Even in rodents, protracted development of the newlyborn neurons contributes to long-term plasticity of the hippocampus, after neurogenesis has declined to low levels (Cole et al., 2020). Studies in that sense, conceived out of the traditional box of “stem cell-driven adult neurogenesis” and opened to explore the possible alternative forms of plasticity designed by nature, are urgently needed.

## Data Availability Statement

The original contributions presented in this study are included in the article/supplementary material, further inquiries can be directed to the corresponding author/s.

## Author Contributions

LB conceived and wrote the manuscript. MG contributed to the research behind the content of the manuscript and to the writing. Both authors contributed to the article and approved the submitted version.

## Conflict of Interest

The authors declare that the research was conducted in the absence of any commercial or financial relationships that could be construed as a potential conflict of interest.

## Publisher’s Note

All claims expressed in this article are solely those of the authors and do not necessarily represent those of their affiliated organizations, or those of the publisher, the editors and the reviewers. Any product that may be evaluated in this article, or claim that may be made by its manufacturer, is not guaranteed or endorsed by the publisher.
